# Circular Bioeconomy: Novel Processes and Materials for Food Preservation

**DOI:** 10.3390/foods12234341

**Published:** 2023-12-01

**Authors:** Sergio Torrres-Giner, Amparo Chiralt, Chelo González-Martínez

**Affiliations:** University Institute of Food Engineering—FoodUPV, Universitat Politècnica de València (UPV), Camino de Vera s/n, 46022 Valencia, Spain; dchiralt@tal.upv.es (A.C.); cgonza@tal.upv.es (C.G.-M.)

Food preservation is a set of procedures and resources aimed at blocking the action of external and internal agents that may alter the original characteristics of food. It has implications regarding aspects of food quality and food safety since it delays the deterioration of organoleptic and nutritional properties and prevents toxicity and contamination. Alteration agents can be grouped into physical (e.g., mechanical damage, temperature, humidity, air, and light), chemical (e.g., browning or rancidity reactions), and biological (e.g., enzymes, macro-, and microorganisms) agents. The food industry has traditionally made use of a combination of different processes and materials to ensure food preservation. These techniques have been classically grouped into two methods, those based on the use of temperature (e.g., pasteurization, sterilization, refrigeration, and freezing) and non-thermal methods (e.g., dehydration and drying processes, the application of electromagnetic waves, radio frequency, salting, curing, acidification, pickling, glazing, smoking, etc.). However, in the 21st century, food preservation technology is facing two major challenges, namely the requirement to reduce food waste and to use more sustainable packaging materials. On the one hand, according to the United Nations Food and Agricultural Organization (FAO), approximately 1/3 of the total food produced worldwide was lost or wasted in the supply chain. This represents 1.3 billion tons of food with direct costs of nearly USD 750 billion yearly. Aside from its economic impact, food waste is a potent greenhouse gas emitter (mainly methane) contributing to environmental pollution. Moreover, the fast-growing world population is accelerating the increased demand for food, and, subsequently, the larger quantity of food production with its increased generation of food wastes. On the other hand, over 85% of food is distributed processed and/or packaged in the retail market, where plastic materials are present in more than 50% of single-use packaging and 40% of the total. This accounts for an annual plastic consumption of approximately 380 million tons, which is based on a linear economy model where 95% of the plastic packaging material value, that is, $80–120 billion annually, is lost after the first short-use cycle. Therefore, both food waste and plastic food packaging pose a challenge not only from an environmental but also from an economic point of view. 

From the above, crafting methods of valorizing food waste and reducing the environmental impact of food plastic packaging are both crucial for developing a sustainable productive model and for achieving the United Nations (UN) Sustainable Development Goals (SDGs) of 2030. For the implementation and achievement of these sustainable development policies, the valorization of food waste under the biorefinery framework together within the circularity of processes and products have gained momentum in recent years. Thus, the shifting of these policies and regulations on how to mitigate and/or utilize food wastes has forced the development of the so-called Circular Bioeconomy, which is now becoming an integral part of industrial green technological processes. It is based on a combination of the principles of both Bioeconomy and Circular Economy, in which biomass and residues are originally regarded as a novel source of raw materials, whereas, at the same time, end products follow a closed model where no waste is generated and the value is recovered. Thus, in the frame of food preservation, the concept of the Circular Bioeconomy can be described as the production of materials and additives from food waste and biomass in a sustainable and integrated/cascaded manner while generating zero waste. Food wastes include both the edible and non-edible parts of food that are generated throughout the whole food supply chain, which are thus potential sources of biopolymers and natural additives. Biopolymers, such as proteins, carbohydrates, and biopolyesters, have special advantages in the domain of biodegradable packaging materials. Moreover, these biopolymers can be formulated with antioxidant and antimicrobial compounds extracted from food waste to develop active and bioactive packaging systems that can contribute to the extension of food shelf life. This Special Issue aims to compile some of the most recent advances in the processes and materials dealing with the valorization of agricultural and food wastes as well as the development of innovative sustainable solutions to improve food preservation and reduce food waste.

In the Circular Bioeconomy context, new food packaging technologies based on biodegradable polymers formulated with natural additives should be developed. As described in the review of Baghi et al. [1], nano- and microcapsules of natural antioxidant and antimicrobial additives can be successfully incorporated into biodegradable polymers to protect and extend the shelf life of perishable foods. Nano- and microencapsulation processes also increase the bioavailability and solubility of these active compounds, providing controlled release and targeted delivery. Furthermore, these additives can additionally improve the thermal and chemical stability of biopolymers. Following this trend, a thymol (TO)-rich activated carbon (AC) nanohybrid structure was prepared by Giannakas and coauthors [2]. This nanohybrid material was, thereafter, extruded with low-density polyethylene (LDPE) to develop novel active packaging films. The authors observed that loadings of 15 wt% of the nanohybrid material improved the barrier properties of LDPE, exhibiting 230% and 1928% higher barriers to water vapor and oxygen, respectively. Moreover, the resultant active film presented high antioxidant activity, showing 44.4% in the 2,2-diphenyl-1-picrylhydrazyl radical (DPPH) assay, a low thymol release rate (k_2_ ≈ 1.5 s^−1^), and high antibacterial activity, resulting in a 2-day extension of fresh pork fillet shelf life. Nevertheless, it is also worth noting that these research works highlighted the current restriction of the application of encapsulated antimicrobial and antioxidant agents in biopolymers for active packaging applications by European regulations for food-contact materials and safety aspects. It was envisaged that the application of nanotechnology in active food packaging will be promoted by the elimination of legislative restrictions and the creation of a unique global organization. At the same time, greater control and monitoring of nanomaterials as well as risk assessment and public consumer familiarization will be required for the acceptance of such an innovation.

In addition, the scientific community is undertaking strong efforts for the utilization of food-processing by-products as a highly sustainable resource to produce food packaging materials and active and bioactive compounds. Multiple research groups are currently engaged in the complete valorization of food wastes into alternative feedstocks for monomers and intermediates to produce biopolymers and green composites. Some intense research is also being conducted in the extraction and purification of antioxidants, natural preservatives, and antimicrobials to enrich food quality and provide food safety. In this regard, Freitas et al. [3] demonstrated the potential of ultrasound-assisted extraction (UAE) as an eco-friendly and efficient process to extract bioactive compounds from rice straw. The performance of UAE treatments in combination with conventional heating and stirring extraction methods was evaluated in terms of the extraction kinetics of phenolic compounds as well as the antioxidant and antimicrobial activities of the obtained extracts. Higher extract yields and antioxidant activities of the water-soluble phenolic compounds were observed through the application of ultrasound pretreatment followed by thermal treatment. In particular, extracts with improved antioxidant activities were attained after ultrasonication for 30 min followed by thermal treatment in water reflux at 100 °C for 60 min. The latter conditions favored, once the substrate surface exposed to the extraction was effectively increased, the promotion of covalent bond cleavage between the phenolic compounds and the lignocellulosic fraction. In another example of valorization, Pavon et al. [4] obtained pine resin and gum rosin derivatives and then used them as natural low-cost additives, such as stabilizers, compatibilizers, and/or plasticizers by. Pine resin and gum rosin, the non-volatile fraction of pine resin, are exudated from conifers and tapping pine trees. The collection of these secretions has recently resurged due to cleaning activities being undertaken to reduce fire risk. The authors showed the benefit of incorporating gum rosin and four gum rosin derivatives into thermoplastic starch (TPS) via melt extrusion. It was determined that loadings of 10 wt% of pine resin derivatives can stiffen the structure of TPS from −100 °C to 40 °C, suggesting a good filler-to-matrix cohesion in this temperature range. Moreover, all the biopolymer/gum rosin formulations disintegrated in less than 90 days under composting conditions. These sustainable materials were proposed to be applied for obtaining rigid packaging materials that do not suffer plastic deformation at room temperature and are fully bio-based and biodegradable and thus aligned with the principles of Circular Bioeconomy.

Also following the Circular Bioeconomy approach, lignocellulosic materials obtained from processing by-products and wastes of the food and agroforestry industries are also gaining more attention as cost-effective fillers in biopolymer-based composites due to their natural origin and favored biodegradability. For instance, Terroba-Delicado et al. [5] valorized spent coffee grounds (SCGs), a type of waste generated during the ‘fruit-to-cup’ transformation of coffee beans. In this research, SCGs obtained from spent coffee grains used in the liquor industry remarkably increased the ductility of compostable polylactide (PLA) pieces. Moreover, the simultaneous addition of chemically modified oligomers of lactic acid (OLAs) contributed to improving the impact strength, and, more notably, the tensile strength of PLA. Thus, the resultant green composite pieces with enhanced mechanical performance are great candidates for food packaging and disposable articles. As shown by Mellinas et al. [6], the cocoa bean shell extract, which is obtained from the residues of the chocolate production process, also represents a great opportunity for waste valorization. The authors developed active films of pectin reinforced with these food waste derived fillers and zinc oxide/zinc nanoparticles (ZnO/Zn-NPs), reporting that both the extract and nanoparticles showed high compatibility with the carbohydrate matrix. As a result, loadings of 3 wt% of nanoparticles enhanced pectin’s barrier to oxygen by 50% and the screen to ultraviolet (UV) radiation reached 98%. Moreover, the photocatalytic activity of the nanoparticle-containing pectin films was demonstrated, showing photodegradation efficiency values of nearly 90% after 60 min, which are all essential properties for the packaging of food that is highly sensitive to oxidative degradation. 

Finally, the novel packaging materials that are currently being developed according to the Circular Bioeconomy’s principles should also provide shelf-life extension to reduce food waste. This was demonstrated, for instance, by Bugatti et al. [7], who developed active cellulose-based trays to preserve ready-to-eat pasta. These trays were made of cellulose coupled with cellulose acetate and coated with layered double hydroxide (LDH) nanofiller containing 4-hydroxybenzoate. The authors evaluated the organoleptic characteristics as well as mold and bacterial growth for up to 30 days of storage at 4 °C, showing that the shelf life of the cooked pasta with tomato sauce was improved. In another study, Hernández-García et al. [8] studied the food packaging performance of films of poly(3-hydroxybutyrate-*co*-3-hydroxyvalerate) (PHBV) reinforced with atomized microfibrillated cellulose (MFC). To this end, the green composite films were applied as lids in trays to package minced pork meat and sunflower oil. It was observed that the PHBV/MFC films successfully preserved the physicochemical and microbiological quality of pork meat for one week of storage at 5 °C, whereas these also prevented sunflower oil oxidation in accelerated oxidative storage conditions for 21 days. The quality and shelf life of pork meat were also evaluated in another research study using multilayer PLA films [9]. The packaged meat fillets were effectively preserved for 7 days in PLA, showing values of food quality and safety in the range of commercial high-barrier multilayers, which are currently based on structures made of non-biodegradable polymers that are extremely difficult to recycle. Finally, Sun et al. [10] demonstrated that films of poly(L-lactide-*co*-butylene fumarate) (PLBF) and poly(L-lactide-*co*-glycolic acid) (PLGA) can successfully extend the shelf life and maintain the quality during cold storage of white mushrooms (*Agaricus bisporus*). These copolymers have the advantage to modify the oxygen (O_2_) and carbon dioxide (CO_2_) ratio of PLA, resulting in lower permeability values. In particular, the copolymer PLBF film was able to offer a shelf life of up 15 days, which was achieved by creating an optimal gas composition for mushroom preservation, that is, 0.27–0.11% O_2_ and 6.33–7.92% CO_2_. This new biopolymer packaging material significantly reduced respiration metabolism, membrane lipid peroxidation, and postharvest senescence.
